# Rim-to-Disc Ratio Outperforms Cup-to-Disc Ratio for Glaucoma Prescreening

**DOI:** 10.1038/s41598-019-43385-2

**Published:** 2019-05-08

**Authors:** J. R. Harish Kumar, Chandra Sekhar Seelamantula, Yogish Subraya Kamath, Rajani Jampala

**Affiliations:** 1 Department of Electrical Engineering, Indian Institute of Science, Bangalore, 560012 India; 2 Department of Electrical and Electronics Engineering, Manipal Institute of Technology, Manipal Academy of Higher Education, Manipal, 576104 India; 3 Department of Ophthalmology, Kasturba Medical College, Manipal Academy of Higher Education, Manipal, 576104 India

**Keywords:** Diagnosis, Biomedical engineering, Electrical and electronic engineering

## Abstract

We present a novel and fully automated fundus image processing technique for glaucoma prescreening based on the rim-to-disc ratio (RDR). The technique accurately segments the optic disc and optic cup and then computes the RDR based on which it is possible to differentiate a normal fundus from a glaucomatous one. The technique performs a further categorization into normal, moderate, or severely glaucomatous classes following the disc-damage-likelihood scale (DDLS). To the best of our knowledge, this is the first engineering attempt at using RDR and DDLS to perform glaucoma severity assessment. The segmentation of the optic disc and cup is based on the *active disc*, whose parameters are optimized to maximize the local contrast. The optimization is performed efficiently by means of a multiscale representation, accelerated gradient-descent, and Green’s theorem. Validations are performed on several publicly available databases as well as data provided by manufacturers of some commercially available fundus imaging devices. The segmentation and classification performance is assessed against expert clinician annotations in terms of sensitivity, specificity, accuracy, Jaccard, and Dice similarity indices. The results show that RDR based automated glaucoma assessment is about 8% to 10% more accurate than a cup-to-disc ratio (CDR) based system. An ablation study carried out considering the ground-truth expert outlines alone for classification showed that RDR is superior to CDR by 5.28% in a two-stage classification and about 3.21% in a three-stage severity grading.

## Introduction

The optic disc is a small blind spot on the surface of the retina and does not contain any photoreceptors. Millions of nerve fibers run from the retina to the optic nerve and converge at the optic disc. The optic disc has three distinct regions: a central white depression called the *cup*, a peripheral ring-shaped region called the *neuroretinal rim*, and the *optic nerves*. Glaucoma is caused by high intraocular fluid pressure created by the abnormal production or drainage of the aqueous humor circulating between the cornea and the lens inside the eye. Glaucoma is an optic neuropathy^1^ – it is a chronic, irreversible, and progressive eye disease that involves loss of retinal nerve fiber layer in a characteristic pattern^2^. It is a leading cause of visual disability and a global public health issue. According to the World Health Organization (WHO), glaucoma is the second leading cause of blindness, after cataract. It is estimated that, by 2020, the total number of glaucoma cases worldwide would be well over 80 million^3^. Early detection and diagnosis of glaucoma requires a careful and comprehensive eye examination, and modern digital technology could aid in rapid assessment.

Since glaucoma alters the optic disc topography, commonly known as *cupping*, and associated loss of the visual field, it is treated based upon the structural appearance of the optic disc and its function by means of a fundus image. The various forms of imaging such as Heidelberg retinal tomography (HRT), scanning laser polarimetry (SLP), and optical-coherence tomography (OCT), permit quantitative measurement of the optic disc and retinal nerve fiber layer (RNFL) structure^4^. Optical-coherence tomography detects damage of the RNFL, whereas HRT characterizes changes in the optic nerve topography. Giaconi *et al*. observed that these imaging modalities complement each other very well, but their sensitivity and specificity are not adequate from diagnostic perspective^4^. Of late, several portable and cost-effective fundus imaging devices and fundus-on-phone (FOP) devices have become available. It is with such devices in mind that we address the goal of developing robust image processing techniques for glaucoma assessment with severity grading and create easy-to-use software that would be suitable for deployment in mass screening programs.

**Figure 1:**
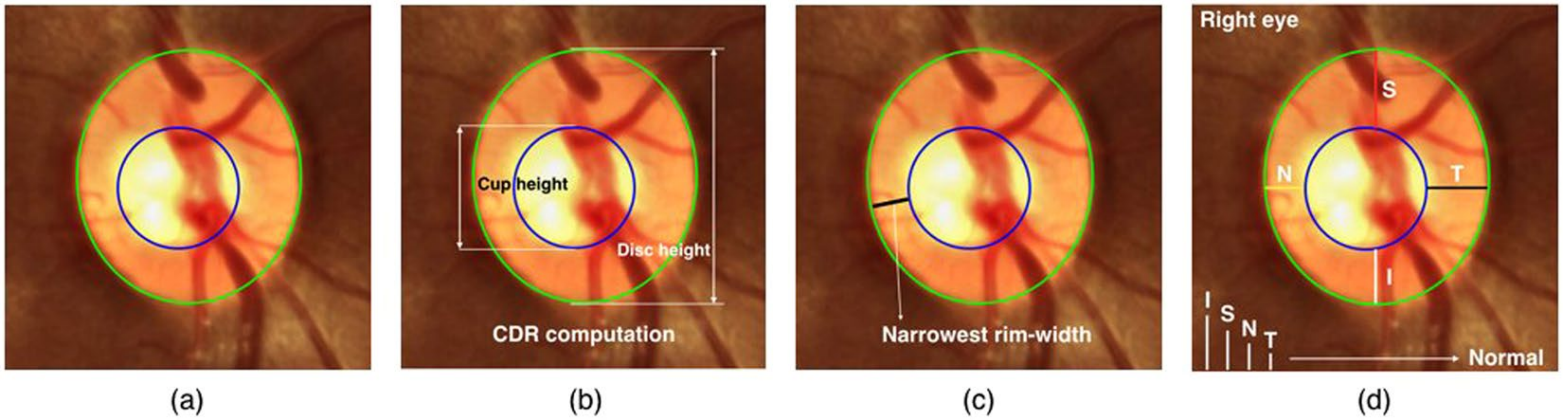
(**a**) Neuroretinal rim (the region between the optic disc and cup); Clinically relevant parameters for glaucoma assessment: (**b**) Cup-to-disc ratio, (**c**) Rim-to-disc ratio, and (**d**) ISNT.

To identify the onset of glaucoma and follow-up on its progression, it is important to quantify the size and shape of the optic disc, cup, and neuroretinal rim^4,5^. The cup-to-disc ratio (CDR) proposed by Armaly *et al*.^6^, rim-to-disc ratio (RDR) based on disc-damage-likelihood-scale (DDLS) proposed by Spaeth *et al*.^7^, and a check for the pattern of neuroretinal rim-widths: Inferior ≥ Superior ≥ Nasal ≥ Temporal (ISNT), proposed by Jonas *et al*.^8^ are important parameters that the ophthalmologists rely on. However, CDR has been the most popular measure that the biomedical engineering community has relied on. The goal of this paper is to assess the importance of the other measures such as RDR and ISNT rim-widths^9–13^ in comparison with CDR and determine which one offers a more reliable and accurate assessment. Automatic determination of these parameters is important as manual annotation of the optic disc and cup and subsequent computation of the parameters is a tedious and time-consuming process. Moreover, fatigue may result in variability and loss of consistency in the manual outlining process. Therefore, automated outlining of the optic disc and cup and determination of CDR/RDR/ISNT rim-widths (cf. Fig. 1) become crucial^6,7,14^.

**Figure 2:**
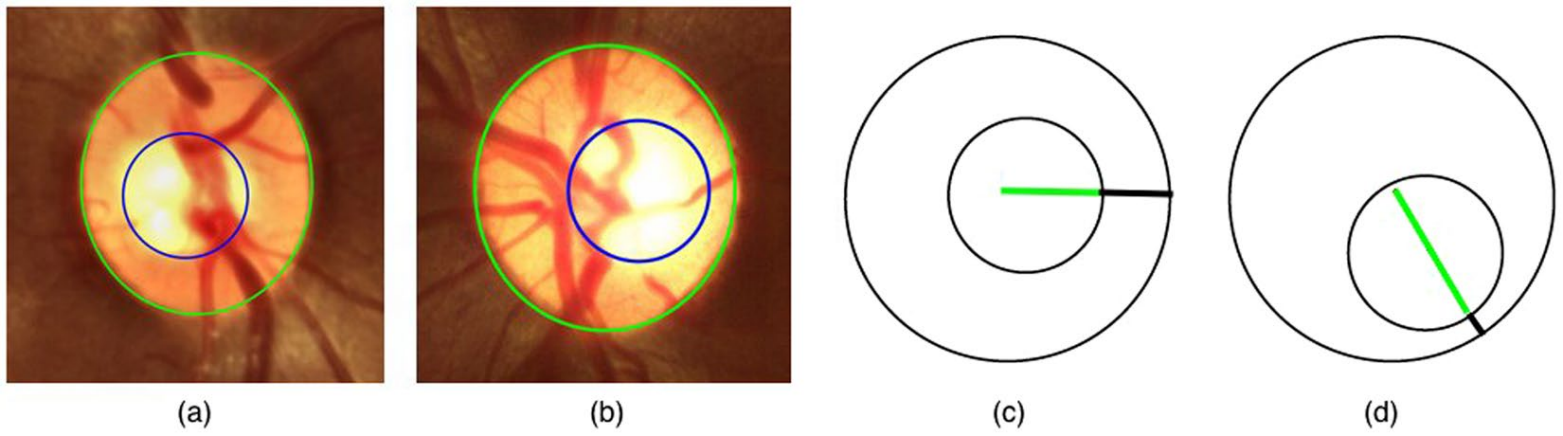
(**a**) Nearly concentric optic disc and cup but non-glaucomatous. (**b**) Eccentric disc and cup but glaucomatous. (**c**,**d**) Two optic disc and cup drawings with identical CDR but with unequal rim-width and with (**c**) being non-glaucomatous, and (**d**) being glaucomatous.

During the past decade, efficient methods have been developed to segment the optic disc, but optic cup segmentation has not received as much importance. A review of the literature on segmentation is presented in Section S1 of Supporting Information. Since 1960s, CDR has been the most commonly used parameter for measuring the relative amount of *cupping*. However, it has two limitations: (i) it does not reflect the size of the optic disc and the position of the cup; and (ii) it does not take into account the amount of loss of nerve fibers in the neuroretinal rim, which directly determines the loss of the visual field. An optic cup that is eccentric is far more likely to be glaucomatous than a concentric one^15–18^. Figure 2 serves as an illustration to show that the CDR is not reliable in such scenarios. From a clinical perspective, ISNT has been shown to be inaccurate for performing glaucoma assessment^12,13^. The RDR parameter with DDLS takes into account the size of the optic disc, position of the optic cup, the narrowest neuroretinal rim-width, and thereby accurately captures the disc abnormality. Rim-to-disc ratio also has the advantage that it overcomes the effect of the optic disc size, which is highly variable in a population^19^. The DDLS has been validated against visual field loss and found to be more reproducible and reliable than HRT^7,15–18^. Abdul Majid *et al*. conducted a clinical study on 149 patients and categorized them as normal, glaucoma suspect, or with glaucoma, employing a DDLS based grading. They established that the DDLS has the best predictive power and showed that it exhibits a close correlation with visual field, CDR, and OCT parameters^20^. A related study by Pahlitzsch *et al*. on patients with preperimetric primary open-angle glaucoma reinforced the predictive power of the DDLS, which makes it an important tool in diagnosing glaucoma^21^. Kara-José *et al*. carried out optic disc clinical evaluation with the DDLS system and the CDR, which showed high accuracy in distinguishing healthy eyes from glaucomatous ones. They showed that the DDLS has moderate-to-strong correlations with most anatomical and functional tests employed for detecting glaucoma^22^. Danesh-Meyer *et al*. conducted a HRT-based clinical study on 110 patients for glaucoma prescreening and evaluated the relationship between the DDLS score, global and sectoral optic disc parameters and visual field indices^23^. A recent study by Hornová *et al*. established that evaluation of the optic nerve head with the DDLS has a high correlation with visual field and the data obtained from HRT-II^24^. Spaeth and Reddy compared various forms of optic nerve imaging such as ophthalmoscopy, fundus photography, OCT, HRT, scanning laser polarimetry, and argued that ophthalmoscopy and fundus photography remain the gold standard of imaging^25^. To the best of our knowledge, a fundus-image-based computer-aided technique using RDR and DDLS-based severity grading has not been reported in the literature although there is strong clinical evidence in its favour. The objective of this paper is to precisely fill this engineering gap.

## Methods

**Figure 3:**
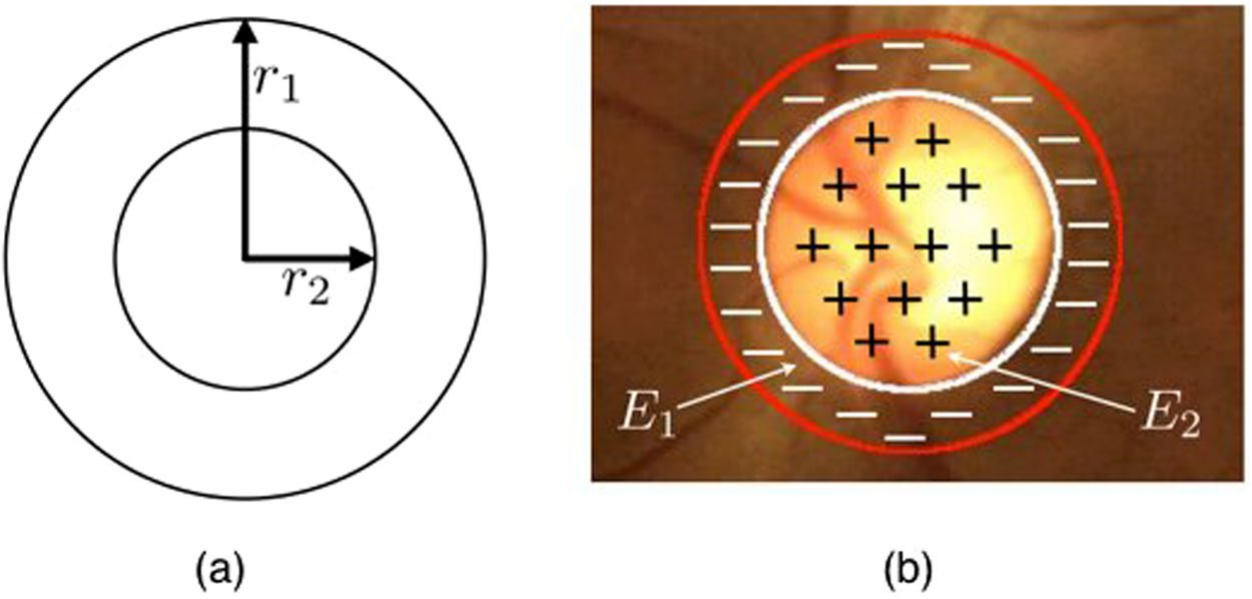
(**a**) Disc template. (**b**) Optimal active-disc fit on the optic disc.

The first step in our approach is the segmentation of the optic disc and cup, which is carried out using the concept of active discs that we introduced recently^26,27^. The segmentation is based on the active disc, which comprises two concentric circular discs centered at the origin and parameterized as follows:

1 $$(\begin{array}{c}{x}_{i}(t)\\ {y}_{i}(t)\end{array})=(\begin{array}{c}{r}_{i}\,\cos \,t\\ {r}_{i}\,\sin \,t\end{array}),$$

for *i* = 1, 2, and $\forall t\in (0,\,2\pi ]\,$, where *r*_*i*_ for *i* = 1, 2 represents the radius of the outer and inner discs, which are set to 1 and $1/\surd 2$, respectively. An example of such a template is shown in Fig. 3(a). The concentric discs with isotropic scaling and translation are given by

2 $$(\begin{array}{c}{X}_{i}\\ {Y}_{i}\end{array})=R(\begin{array}{c}{x}_{i}\\ {y}_{i}\end{array})+(\begin{array}{c}{x}_{c}\\ {y}_{c}\end{array}),$$

where *i* = 1, 2, and (*X*_1_, *Y*_1_), and (*X*_2_, *Y*_2_) are the outer and inner boundaries, respectively, *R* represents the scale parameter and (*x*_*c*_, *y*_*c*_) are the translational parameters, amounting to a total of three degrees of freedom. For brevity of notation, we replace (*x*_*i*_(*t*), *y*_*i*_(*t*)) and (*X*_*i*_(*t)*, *Y*_*i*_(*t*)) with (*x*_*i*_, *y*_*i*_) and (*X*_*i*_, *Y*_*i*_), respectively. The active disc energy is based on a normalized contrast, which considers the area inside the inner disc as the foreground and the annular region as the background. For an image *f*, let *R*_1_and *R*_2_ be the regions enclosed by the outer and inner discs, respectively. The objective function is chosen to be

3 $$\begin{array}{ccc}E & = & \frac{1}{{R}^{2}}({\iint }_{{R}_{1}{\rm{\backslash }}{R}_{2}}f(X,Y)dXdY-{\iint }_{{R}_{2}}f(X,Y)dXdY),\\  & = & \frac{1}{{R}^{2}}({\iint }_{{R}_{1}}f(X,Y)dXdY-2{\iint }_{{R}_{2}}f(X,Y)dXdY),\\  & = & \,\frac{1}{{R}^{2}}({E}_{1}-2{E}_{2}),\end{array}$$

where *E*_1_ and *E*_2_ are the image energies in the regions *R*_1_and *R*_2_, respectively. Optimizing *E* would maximize the contrast between the two regions. The active disc is evolved from an initialization towards the boundary of the optic disc such that the energy is minimized. Figure 3(b) gives an example of the optimal fit contour.

### Initialization

Accurate initialization is essential for reliable segmentation. We develop an automatic initialization technique using a multiscale normalized matched filter, inspired by the pyramidal decomposition method of Lalonde *et al*.^28^. To begin with, we construct a three-level pyramid. At the lowest level, the cross-correlation between the fundus image and a natural optic disc template is computed. The peak in the result is selected for initializing the template at that scale. The peak location (*x*_*p*_, *y*_*p*_) in level 3 is mapped to the image at the top of the pyramid. After localization, the region of interest surrounding the optic disc is chosen for subsequent analysis. The details of the initialization procedure are given in Section S2 of Supporting Information.

### Optic disc segmentation

We use the red channel of the monocular color fundus image for disc segmentation, the reason being that the blood vessels and optic nerve occlusions get de-emphasized compared with the other channels or the grayscale version. Also, the optic disc appears brightest in the red channel, which goes well with our local-contrast-based segmentation strategy. The segmentation is accelerated using a multiscale strategy. The best-fit disc outline is obtained on the coarse-level representation at the bottom of the image pyramid. The converged result is used to determine the initialization for the immediate next finer resolution image. This process is repeated all the way to the top of the pyramid resulting in the final outline. The details of the active disc formulation, optimization and means to enhance computational efficiency are given in Section S3 of Supporting Information.

### Optic cup segmentation

For cup segmentation, we extract the green channel as it has a higher contrast. We use Otsu’s multilevel thresholding algorithm^29,30^ to classify and cluster the pixels corresponding to the optic cup, and then refine it using the active disc. Examples of coarse optic cup segmentation achieved using Otsu’s algorithm are provided in Section S4 of Supporting Information. We found that a four-level thresholding scheme works well for clustering the optic cup pixels. The procedure used to obtain the optic cup boundary is similar to that explained in Section S3 of the Supporting Information.

### Performance metrics

Sensitivity, specificity, accuracy, Jaccard, and Dice similarity indices are the standard comparison metrics^31,32^ used in medical image segmentation. They are based on true positives (*TP*), true negatives (*TN*), false positives (*FP*), and false negatives (*FN*). Sensitivity is the proportion of positives that are correctly identified as positives, whereas specificity is the proportion of negatives that are correctly identified as negatives. Accuracy is the degree of closeness between the algorithm segmentation and the expert clinician segmentation.

$${\rm{S}}{\rm{e}}{\rm{n}}{\rm{s}}{\rm{i}}{\rm{t}}{\rm{i}}{\rm{v}}{\rm{i}}{\rm{t}}{\rm{y}}=\frac{TP}{TP+FN};{\rm{S}}{\rm{p}}{\rm{e}}{\rm{c}}{\rm{i}}{\rm{f}}{\rm{i}}{\rm{c}}{\rm{i}}{\rm{t}}{\rm{y}}=\frac{TN}{TN+FP};{\rm{A}}{\rm{c}}{\rm{c}}{\rm{u}}{\rm{r}}{\rm{a}}{\rm{c}}{\rm{y}}=\frac{TP+TN}{TP+TN+FP+FN}.$$

The Jaccard and Dice similarity indices are defined as Jaccard $=\frac{|A\cap M|}{|A+M|}$ and Dice $=\frac{2|A\cap M|}{|A|+|M|}$, respectively, where *A* and *M* represent the optic disc or cup region segmented by the algorithm and the manual segmentation provided by the clinician, respectively.

### Parameters of clinical relevance

The clinically relevant parameters that we shall consider are: CDR^6^, RDR followed by DDLS^7^, and a check for the ISNT pattern of neuroretinal rim-widths^8^. It turns out that the geometry of the disc and cup outlines obtained after segmentation provides a precise mathematical basis for determining the smallest neuroretinal rim-width. We estimate the vertical height of the optic disc and cup and also narrowest rim-width for the computation of CDR, RDR, and inferior, superior, nasal, and temporal rim-widths for checking the ISNT pattern. We follow the International Classification of Diseases (ICD-9) rule^33^ based on CDR for a three-stage assessment of glaucoma severity as follows: normal (0 ≤ CDR ≤ 0.5); moderate (0.5 ≤ CDR ≤ 0.8); and severely glaucomatous (0.8 ≤ CDR ≤ 1).

### Severity assessment of glaucoma using DDLS based on RDR

**Figure 4:**
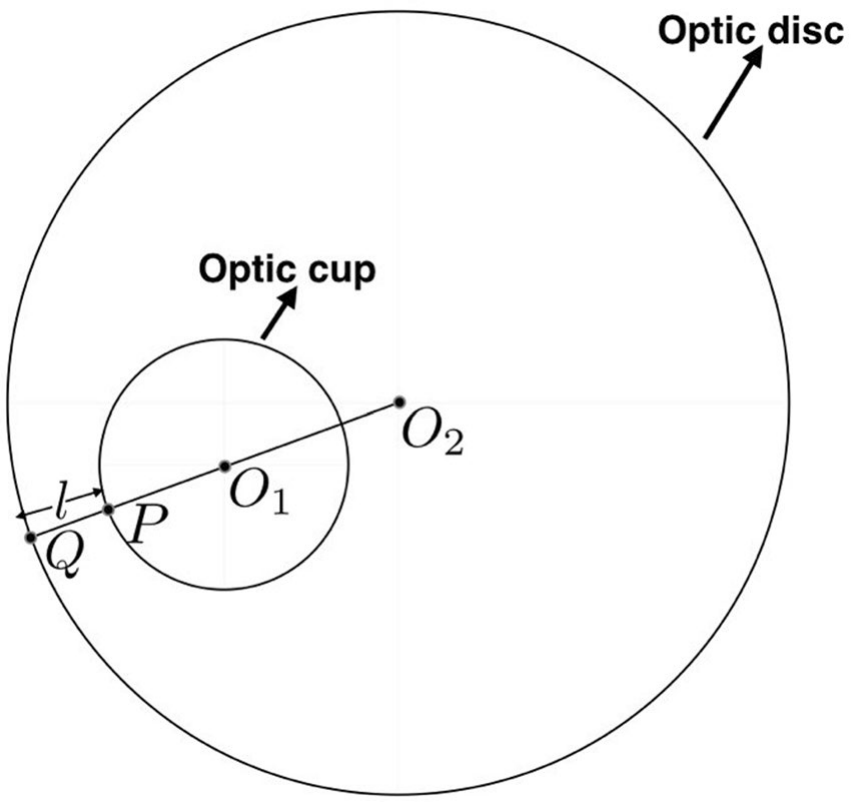
Determination of the narrowest rim-width. *O*_1_ and *O*_2_ denote the centers of the optic cup and disc, respectively. The narrowest rim-width is *l*.

The DDLS takes into account the optic disc size and the optic cup location and is a reliable indicator of the visual field loss. The smallest neuroretinal rim-width is an important parameter for DDLS-based glaucoma stage analysis. The reliability of DDLS has been proved by Henderer *et al*.^16,17^ and its clinical relevance compared with CDR and HRT for glaucoma diagnosis has been established by Meyer *et al*.^18^. The smallest neuroretinal rim-width can be obtained by considering the geometry of two non-concentric circles one fully inside the other as depicted in Fig. 4. Circles with centers *O*_1_ and *O*_2_ represent the optic cup and disc, respectively. It can be established geometrically that the line joining the centers when extended to the periphery of the outer disc determines the smallest neuroretinal rim-width. Specifically, with respect to Fig. 4, the line segment *PQ* gives the desired rim-width. Based on the narrowest rim-width, glaucomatous conditions can be categorized into 8 or 10 stages as given in Table 1. We consider the following three-stage classification in accordance with the modified rule proposed by Spaeth *et al*.^15^ and Henderer *et al*.^16^, which has now become an internationally accepted guideline:

1. Not definitely damaged (normal);
2. Asymptomatic glaucoma damage (moderate); and
3. Glaucomatous disease/disability (severe).

**Table 1:** Classification of glaucomatous condition based on the narrowest rim-width or RDR using DDLS as recommended by Spaeth *et al*.^15^ and Henderer *et al*.^16^.

DDLS stage (Spaeth *et al*.^7^)	DDLS stage (Spaeth *et al*.^15^ and Henderer *et al*.^16^)	Rim-to-disc ratio (RDR) range	Glaucomatous condition
0 (0a and 0b)	1 and 2	0.30 ≤ RDR ≤ 0.50	Not definitely damaged (Normal)
1	3	0.20 < RDR ≤ 0.29	
2	4	0.10 < RDR ≤ 0.19	
3	5	0.01 < RDR 0.10	Asymptomatic glaucoma damage (Moderate)
4	6	No rim < 45°	
5	7	No rim 45°–90°	
6	8	No rim 90°–180°	Glaucomatous disease/disability (Severe)
7 (7a and 7b)	9 and 10	No rim < 180°	

For comparison, the classification proposed by Spaeth *et al*.^7^ is also shown.

### Database validation

In this section, we examine the performance of the proposed technique on several retinal fundus image databases varying in size, resolution, contrast, and illumination. The publicly available databases used are Messidor^34^, Drishti-GS^35^, DiaretDB1^36^, Drive^37^, and Drions-DB^38^, containing 1200, 101, 89, 40, and 110 retinal fundus images, respectively. The locally obtained databases are from Forus Health Pvt. Ltd.^39^ and Bosch Eye Care Solutions^40^ containing 126 and 60 retinal fundus images, respectively. This amounts to a total of 1726 images. The Forus desktop fundus-imaging camera results in a four-megapixel image, whereas the Bosch handheld device produces images at five-megapixel resolution. The Messidor, Drishti-GS, and Drions-DB databases provide the ground-truth outlines for the optic disc. The Drishti-GS database additionally provides the ground-truth outlines for the optic cup. Manual outlines of the optic disc and cup were provided by two ophthalmologists for the images taken from the Forus and Bosch databases as well as for 386 images drawn randomly from the other databases. This amounts to a total of 1597 images for assessing the optic disc segmentation performance and 436 images for assessing the cup segmentation performance. According to the expert assessment, out of the 436 images, 358 are normal and 78 are glaucomatous considering a two-stage classification. Considering a three-stage severity grading gave rise to 309 normal, 118 moderately glaucomatous, and 9 severely glaucomatous cases.

### Software

The proposed approach has been implemented in Java as an ImageJ plugin^41,42^. A batch-processing ImageJ macro was developed to automatically handle all the images in a database. The performance evaluation was carried out on Mac OS X 2.7 GHz, Intel Core i5 machine. We have also developed iOS and Android apps that can be easily integrated into modern-day smartphone-based glaucoma-prescreening devices. Screenshots of the iOS app are shown in Section S5 of Supporting Information.

## Results

**Figure 5:**
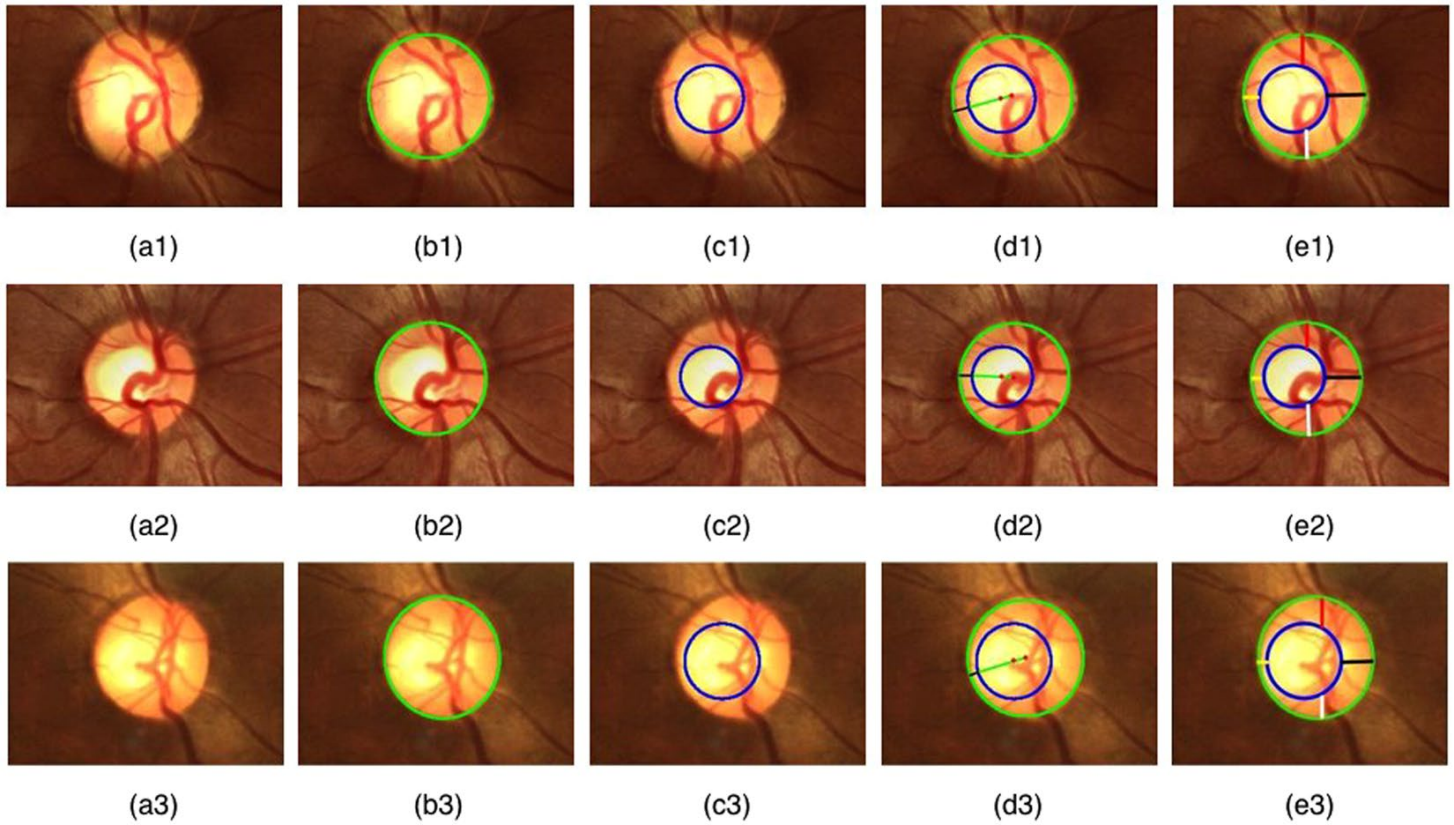
An illustration of the segmentation performance of the proposed method. (**a1**–**a3**) Input fundus images. (**b1**–**b3**) Optic disc outlines (green). (**c1**–**c3**) Optic cup outlines (blue). (**d1**–**d3**) Narrowest rim-width in black color. (**e1**–**e3**) Inferior, superior, nasal, and temporal rim widths in white, red, yellow, and black colors, respectively.

**Figure 6:**
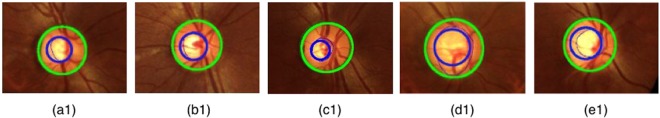
A comparison of the algorithm outlines (thick contours) with the expert outlines (thin contours).

The performance of the proposed multiscale normalized matched filtering technique is given in Table 2. We observe that the localization accuracy is at 100% for four out of seven databases, and greater than 97% for the remaining. The low processing time of 0.32 seconds per image is a direct advantage of employing the multiscale strategy. The optic disc and cup outlines are shown in Fig. 5 for some representative images taken from the databases. Figure 6 shows the algorithm outlines in comparison with the expert outlines. The segmentation performance of the proposed method and the average time taken are summarized in Tables 3 and 4. A comparison of the performance of the proposed method with the state-of-the-art techniques is shown in Section S6 of Supporting Information. The performance metrics indicate that the proposed technique gives outlines that have a high degree of agreement with the expert markings over several databases. Tables 5 and 6 show the performance of the RDR in comparison with CDR based on ICD-9 rule and ISNT for two-stage and three-stage glaucoma classification, respectively. The overall classification accuracy (OCA) over a database is defined as the ratio of the number of images correctly classified to the total number of images used for evaluation from that database. The OCA is higher for RDR based assessment compared with CDR and ISNT. Specifically, the accuracy of RDR is superior to CDR by 7.8% for a two-stage classification and 9.64% for a three-stage severity grading.

**Table 2:** Optic disc localization accuracy of the proposed method.

Algorithm	Database and # of images used	Localization accuracy in %	Average detection time per image in seconds
Proposed method	Drishti-GS (101)	99.01	0.32
	Drions-DB (110)	100.00	
	Drive (40)	100.00	
	DiaretDB1 (89)	97.75	
	Messidor (1200)	99.33	
	Forus (126)	100.00	
	Bosch (60)	100.00

**Table 3:** Performance assessment of optic disc segmentation on various databases.

Database and number of images used	Sensitivity	Specificity	Accuracy	Jaccard index	Dice index	Average time taken per image in seconds
Drishti-GS (101)	0.9962	0.9698	0.9732	0.8707	0.9282	7.35
Drions-DB (110)	0.8410	0.9960	0.9850	0.8341	0.9062	4.45
Messidor (1200)	0.8840	0.9894	0.9882	0.7858	0.8760	6.34
Forus (126)	0.9457	0.9957	0.9911	0.9319	0.9642	6.22
Bosch (60)	0.9649	0.9994	0.9968	0.9109	0.9531	6.35

**Table 4:** Performance assessment of optic cup segmentation on various databases.

Database and number of images used	Sensitivity	Specificity	Accuracy	Jaccard index	Dice index	Average time taken per image in seconds
Drishti-GS (50)	0.8589	0.9698	0.9690	0.6143	0.7532	5.46
Drions-DB (100)	0.6175	0.9979	0.9884	0.5898	0.7300	3.54
Messidor (100)	0.8357	0.9883	0.9823	0.5911	0.7303	4.35
Forus (126)	0.7466	0.9952	0.9867	0.6801	0.8027	4.12
Bosch (60)	0.7584	0.9959	0.9917	0.5804	0.7213	4.28

**Table 5:** Ground-truth and performance comparison for two-stage glaucoma classification.

Database and number of images used	Ground truth		RDR-based results			CDR-based results			ISNT-based results		
	N	G	N	G	OCA	N	G	OCA	N	G	OCA
Drishti-GS (50)	16	34	12	38	43/50	21	29	40/50	08	42	37/50
Drions-DB (100)	90	10	88	12	85/100	79	21	80/100	70	30	71/100
Messidor (100)	92	08	91	09	91/100	82	18	80/100	75	25	73/100
Forus (126)	100	26	99	27	124/126	105	21	110/126	86	40	100/126
Bosch (60)	60	00	59	01	59/60	58	02	58/60	53	07	50/60
**Consolidated** (**436 images**)	**358**	**78**	**349**	**87**	**402/436** (**92.20%**)	**345**	**91**	**368/436** (**84**.**40%**)	**292**	**144**	**331/436** (**75**.**92%**)

(N – Normal; G – Glaucomatous; OCA – Overall Classification Accuracy).

**Table 6:** Ground-truth and performance comparison for three-stage glaucoma classification.

Database and number of images used	Ground truth			RDR-based results (DDLS)				CDR-based results (ICD-9)			
	N	Mod	S	N	Mod	S	OCA	N	Mod	S	OCA
Drishti-GS (50)	13	32	05	19	31	00	42/50	07	42	01	36/50
Drions-DB (100)	78	22	00	77	20	03	82/100	73	25	02	78/100
Messidor (100)	58	41	01	60	40	00	84/100	52	47	01	70/100
Forus (126)	100	23	03	97	27	02	121/126	88	37	01	106/126
Bosch (60)	60	00	00	59	01	00	59/60	56	04	00	56/60
**Consolidated** (**436 images**)	**309**	**118**	**09**	**312**	**119**	**05**	**388/436** (**89%**)	**312**	**119**	**05**	**346/436**(**79**.**36%**)

(N – Normal; Mod – Moderate; S – Severe; OCA – Overall Classification Accuracy).

In order to remove the influence of the segmentation method on the claim, we conducted an ablation study where we also considered the classification performance purely based on the expert outlines (Tables 7 and 8). From these results, we observe that the RDR-based two-stage classification is 5.28% superior to the CDR-based one. In the case of a three-stage severity grading, the accuracy improvement offered by RDR over CDR is 3.21%.

**Table 7:** Expert-outline-based two-stage classification performance.

Database and number of images used	RDR-based assessment			CDR-based assessment		
	Sensitivity	Specificity	Accuracy	Sensitivity	Specificity	Accuracy
Drishti-GS (50)	0.9706	0.9375	0.9600	0.9412	0.7500	0.8800
Drions-DB (100)	1.0000	0.9560	0.9600	1.0000	0.9121	0.9200
Messidor (100)	1.0000	0.9560	0.9600	0.8889	0.8791	0.8800
Forus (126)	0.9630	0.9697	0.9683	0.8889	0.9091	0.9048
Bosch (60)	—	1.0000	1.0000	—	1.0000	1.0000
Consolidated (436 images)	0.9625	0.9663	0.9656	0.9241	0.9104	0.9128

**Table 8:** Expert-outline-based three-stage classification performance.

Database and number of images used	RDR-based assessment			CDR-based assessment		
	Sensitivity	Specificity	Accuracy	Sensitivity	Specificity	Accuracy
Drishti-GS (50)	0.8421	0.8333	0.8400	0.8158	0.7500	0.8000
Drions-DB (100)	0.8871	0.8861	0.8800	0.6667	0.8861	0.8400
Messidor (100)	0.8667	0.9091	0.8900	0.8444	0.8909	0.8700
Forus (126)	0.8462	0.9100	0.8968	0.8077	0.8609	0.8492
Bosch (60)	—	1.0000	1.0000	—	1.0000	1.0000
Consolidated (436 images)	0.8538	0.9183	0.8991	0.8000	0.8954	0.8679

The preceding study establishes the superiority of RDR-based glaucoma classification over a CDR-based one.

## Conclusions

In this paper, we developed a new method to perform severity grading of glaucoma based on RDR. We showed that the classification accuracy is higher when one uses the RDR-based DDLS instead of the frequently employed CDR, both in case of automated segmentation as well as expert annotation. From a clinical perspective also, RDR is more representative of the severity of the glaucoma than CDR as it captures the loss of neuroretinal fiber more accurately. The high accuracy is reflected in both two-stage and three-stage glaucoma classification. As far as estimating the RDR is concerned, we deployed a novel active disc template based method operating in a multiscale framework. The optic disc and cup segmentation results were found to be in high agreement with the expert outlines over a large number of images taken from various databases. In keeping with the contemporary and game-changing trend of developing smartphone-based eye-care solutions, we have also developed associated software in Java and Apps for Android and iOS devices, which operate in real-time on the device without the need for processing on a centralized server or in the cloud. These software solutions are particularly attractive for triaging applications using modern day handheld fundus imaging devices, which are heavily deployed for mass-screening purposes in the third-world countries in particular those in south Asia and Africa.

## Supplementary information

Rim-to-Disc Ratio Outperforms Cup-to-Disc Ratio for Glaucoma Prescreening
