# Supplementary material for: Rim-to-Disc Ratio Outperforms Cup-to-Disc Ratio for Glaucoma Prescreening
Source: Sci Rep. 2019 May 8;9:7099. doi: 10.1038/s41598-019-43385-2 (PMC6506519; doi:10.1038/s41598-019-43385-2)
Supplement: Supplementary file 1 — Rim-to-Disc Ratio Outperforms Cup-to-Disc Ratio for Glaucoma Prescreening [file 41598_2019_43385_MOESM1_ESM.pdf]

Rim-to-Disc Ratio Outperforms Cup-to-Disc Ratio for Glaucoma Prescreening

Supporting Information

J. R. Harish Kumar,[1,2*] Chandra Sekhar Seelamantula,[1*] Yogish Subraya Kamath,[3] and Rajani Jampala[3]

[1]Department of Electrical Engineering, Indian Institute of Science, Bangalore-560012, India
[2]Department of Electrical and Electronics Engineering, Manipal Institute of Technology, Manipal Academy of Higher Education, Manipal-576104, India
[3]Department of Ophthalmology, Kasturba Medical College, Manipal Academy of Higher Education, Manipal-576104, India

[*]Corresponding authors: E-mail: harishj@iisc.ac.in, chandrasekhar@iisc.ac.in

S1 Literature Review

S1.1 Optic Disc Segmentation: State-of-the-Art

The first step in a computer-assisted assessment of glaucoma based on Cup-to-Disc Ratio (CDR), Rim-to-Disc Ratio (RDR) computation, or a check for Inferior $\geq$ Superior $\geq$ Nasal $\geq$ Temporal (ISNT) rim-width pattern starting from retinal fundus images, is the localization and segmentation of the optic disc and cup. The problem of optic disc segmentation has received a lot more attention in the literature than optic cup boundary detection although both are equally important problems for glaucoma prescreening. The segmentation of optic disc contour is challenging because of the presence of blood vessels and optic nerve occlusion, and peripapillary atrophy. In certain cases, there are also large exudative lesions, which pose further challenges. In this section, we review important literature on optic disc and cup segmentation.

Foracchia et al.[1] proposed a method based on a model of the geometrical directional pattern of the retinal vascular system, considering the optic disc region as the point of convergence of all vessels. Foracchia et al. fit a model with respect to the entire retinal vascular structure. Morales et al.[2] proposed a method based on mathematical morphology coupled with principal component analysis. The method uses a generalized distance function, a variant of the watershed transformation, the stochastic watershed and geodesic transformations. Carmona et al.[3] used genetic algorithms to locate and segment the optic nerve head. A set of hypothesis points were obtained that exhibited geometric properties and intensity levels similar to the optic nerve head contour pixels. The genetic algorithm was then used to find an ellipse containing the maximum number of hypothesis points in an offset of its perimeter, which then becomes the approximation to the optic nerve head. Mahfouz et al.[4] presented a fast technique to localize the optic disc based on obtaining two projections of certain image features that encode the horizontal and vertical coordinates of the optic disc. The resulting 1-D projections are then searched to determine the location of the optic disc. Aquino et al.[5] proposed a new template-based methodology for segmenting the optic disc. Their method uses morphological and edge detection techniques followed by the circular Hough transform to obtain a circular optic disc boundary approximation. Yu et al.[6] identified the optic disc location using template matching and directional matched filter. Based on the detected location, a fast hybrid level-set algorithm, which combines the region information and edge gradient to drive the curve evolution, is used to segment the optic disc boundary. Giachetti et al.[7] proposed a method that first separates the vasculature, in-paints those areas and finally fits a contour model to the optic disc. Youssif et al.[8] proposed a method based on matching the expected directional pattern of the retinal blood vessels using a matched filter. Illumination equalization and adaptive histogram equalization methods are used as preprocessing steps. The region-based active contour model proposed by Joshi et al.[9] uses statistical information from the background and the foreground region to minimize the energy function to best separate the regions. Muramatsu et al.[10] proposed a pixel classification method for clustering and classifying image pixels as optic disc or background pixels to segment the optic disc using fuzzy c-means and artificial neural networks. Lowell et al.[11] developed Hough transform-based circular or elliptical template matching techniques.

Pallawala et al.[12] used wavelet transform and ellipse fitting for automated optic disc detection and localization. Chrastek et al.[13] used nonlinear filtering, Canny edge detector, and Hough transform for the localization and segmentation of optic disc. Welfer et al.[14] proposed an adaptive morphology-based technique for the segmentation of optic disc. Osareh[15] used morphological processing and template matching for optic disc localization and the gradient vector flow snakes for optic disc segmentation. Walter et al.[16] proposed marker-controlled-watershed-transformation to segment the optic disc. Abdullah et al.[17] employed circular Hough transform, and the grow-cut algorithm to precisely segment the optic disc boundary. Gonzalez et al.[18] extract the retinal vascular tree in the first step and then use the graph-cut technique for the segmentation of optic disc.

Recently, deep convolutional neural networks (CNNs) have been successfully applied to sophisticated tasks such as object detection and segmentation. Sevastopolysky et al.[19] proposed a modified U-Net[20] architecture to generate the optic disc segmentation map. Maninis et al.[21] proposed an architecture based on visual geometry group's 16-layer model (VGG16[22]) with specialized layers for retinal vasculature segmentation and optic disc segmentation.

S1.2 Optic Cup Segmentation: State-of-the-Art

Accurate segmentation of optic cup region is essential in the automatic assessment of glaucoma. The optic cup depth information is a major indicator of the optic cup boundary with two important landmarks such as the pallor and vessel bends. Optic cup segmentation is difficult as the fundus images do not carry direct depth information. Various automated optic cup segmentation methods have been presented in the literature. Joshi et al.[23] proposed a novel, depth discontinuity in the retinal surface-based approach to estimate the optic cup boundary. The optic cup boundary is approximated with a best-fitting circle. Joshi et al.[24] used another method based on vessel bends at optic cup boundary. Vessels are modeled and detected in a curvature space and bends in a vessel are robustly detected using a region of support concept. A reliable subset called r-bends is derived using a multistage strategy and a local spline fitting is used to obtain the desired optic cup boundary. Hatanaka et al.[25] proposed an improved automated optic cup segmentation technique based on detection of blood vessel bends in retinal fundus images. A concentration feature determined from the density gradient detects the blood vessels. The blood vessel bends are detected by tracking the blood vessels from the disc edge to the primary cup edge. Chakravarthy et al.[26] proposed a novel, supervised method for depth-based cup segmentation. Xu et al.[27] followed the super-pixel framework and domain prior to segment the optic cup, where the super-pixel classification task is formulated as a low-rank representation problem with an efficient closed-form solution. Luangruangrong et at.[28] employed a polar space contour detection approach for the segmentation of optic cup.

S1.3 Optic Cup and Disc Segmentation: State-of-the-Art

Few papers in the literature have addressed the problem of both cup and disc segmentation. Cheng et al.[29] employed a super-pixel classification approach. In optic disc segmentation, histograms and center surround statistics are used to classify each super pixel as disc or non-disc. A deformation step using deformable models is used to fine-tune the disc boundary. For optic cup segmentation, in addition to the histograms and center surround statistics, the location information is also included. Xu et al.[30] used deformable snakes to segment the optic disc. The contour deforms to the location with minimum energy, and then self-clusters into edge-point group and uncertain-point group. The method is then extended to detect the optic cup boundary. The contour is initialized by Hough transformation in edge map, and then processed by contour deformation, knowledge-based clustering, and updating. Joshi et al.[31] used optic disc and optic cup boundaries to estimate the relevant disk parameters. A deformable model guided by regional statistics detects optic disc boundary. The optic cup boundary detection is based on the appearance of pallor in Lab color space and the expected optic cup symmetry. Joshi et al.[32] proposed a novel region based active contour model to segment the optic disc. The optic cup segmentation was done by the detection of vessel bends[24] in the optic cup region. Yin et al.[33] proposed a statistical model-based method for the segmentation of optic disc and optic cup from fundus images. The method combines knowledge-based circular Hough transform and a novel optimal channel selection for segmentation of the optic disc and optic cup. Zahoor et al.[34] presented a polar transform based approach for the segmentation of optic disc and cup. Cheng et al.[35] proposed a structure-preserving guided retinal image filtering approach to remove artifacts. They employed deep learning based technique for optic cup segmentation

and sparse learning based technique for cup-to-disc ratio measurement. Winder et al.[36] reviewed the recent literature on preprocessing, localization and segmentation of the optic disc and retinal vasculature, localization of the macula and fovea, and localization and segmentation of retinopathy.

S2 Automatic Localization of the Optic Disc Region

We localize the optic disc region using a normalized matched filter with a manually segmented and subsampled optic disc region as the template. For reasons of computational efficiency and speed, the matched filtering is performed at the lowest level of a four-level pyramid representation of the image and then the location information is propagated to the highest level of the decomposition. The normalization takes care of intensity variations between the image and the template. The normalized matched filter is equivalent to computing a cosine similarity measure and ensures that the localization is unaffected by pathologies such as lesions. The cosine similarity measure ξ between the image f at the jth level of the pyramid $f^{(j)}$ and the template h is given by

$$\xi(x_p, y_p) = \cos\phi = \left\langle \frac{f^{(j)}(x,y)}{\|f^{(j)}(x,y)\|}, \frac{h(x-x_p, y-y_p)}{\|h(x-x_p, y-y_p)\|} \right\rangle,$$

where $\|\cdot\|$ denotes the ℓ_2-norm. The location corresponding to the peak of the cosine similarity measure is considered for initializing the active disc (cf. Figure 1 for an illustration).

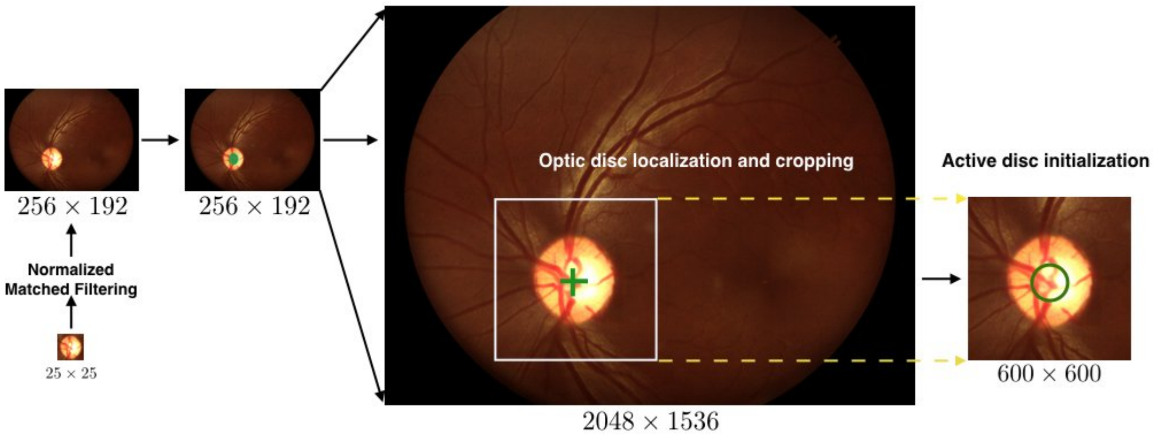

Figure 1: Localization and selection of region of interest and subsequent initialization of the active disc.

S3 Active Disc Formulation and Optimization

The active disc template comprises a pair of concentric outer and inner discs parametrized as:

$$\begin{pmatrix} x_i(t) \\ y_i(t) \end{pmatrix} = \begin{pmatrix} r_i \cos t \\ r_i \sin t \end{pmatrix}, \tag{1}$$

for $i = 1, 2$, and $\forall t \in (0, 2\pi]$, where r_i for $i = 1, 2$ represents the radius of the outer and inner discs which are set to 1 and $1/\sqrt{2}$, respectively. An example of such a circular template is shown in Fig. 2. The factor $1/\sqrt{2}$ is used to make the area of the inner disc equal to that of the annulus, which ensures that the circular active disc is inert in regions of constant intensity. We consider the transformation comprising isotropic scaling and translation given as follows:

$$\begin{pmatrix} X_i(t) \\ Y_i(t) \end{pmatrix} = R \begin{pmatrix} x_i(t) \\ y_i(t) \end{pmatrix} + \begin{pmatrix} x_c \\ y_c \end{pmatrix}, \tag{2}$$

where R is the scale parameter and x_c, y_c are the translation parameters, accounting for a total of three degrees of freedom. Figure 2 illustrates the disc template and the active disc. For brevity of notation, we replace $(x_i(t), y_i(t))$ and $(X_i(t), Y_i(t))$ with (x_i, y_i) and (X_i, Y_i), respectively.

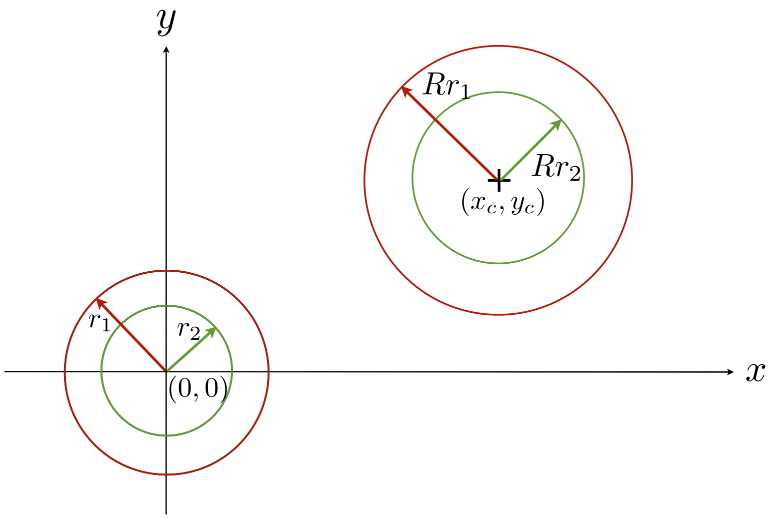

Figure 2: The disc template and its *active* counterpart.

S3.1 Active Disc Energy

The active disc energy is the normalized local contrast function defined as

$$\begin{aligned} E &= \frac{1}{R^2}\left(\iint_{R_1 \setminus R_2} f(X,Y)\,\mathrm{d}X\,\mathrm{d}Y - \iint_{R_2} f(X,Y)\,\mathrm{d}X\,\mathrm{d}Y \right), \\ &= \frac{1}{R^2}\left(\underbrace{\iint_{R_1} f(X,Y)\mathrm{d}X\,\mathrm{d}Y}_{E_1} - 2\underbrace{\iint_{R_2} f(X,Y)\mathrm{d}X\,\mathrm{d}Y}_{E_2} \right), \\ E &= \frac{1}{R^2}(E_1 - 2E_2), \end{aligned} \tag{3}$$

where E_1 and E_2 are the energies of the outer and inner discs, respectively. $f(X, Y)$ is the coordinate-axes-transformed image of $f(x, y)$ and is given by

$$f(X, Y) \leftarrow f\left(\frac{X - x_c}{R}, \frac{Y - y_c}{R}\right).$$

Minimizing E with respect to the parameters $\{R, x_c, y_c\}$ enables the active disc to lock on to the optic disc, which is brighter than its immediate surroundings. The area based normalization ensures that the tightest fit outline is obtained. A typical optic disc image, the initialization and the converged active disc are shown in Figure 3.

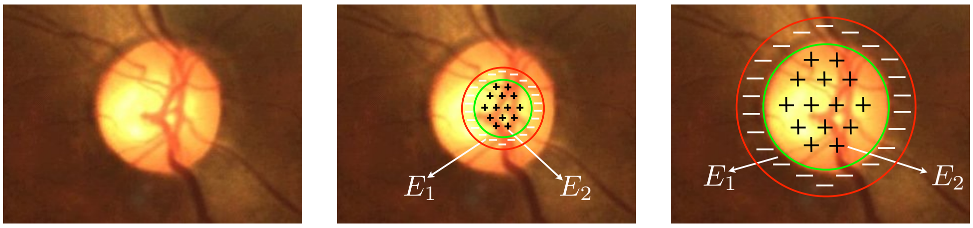

(a) (b) (c)

Figure 3: (a) Optic disc image; (b) Active disc initialization; and (c) Optimized active disc.

S3.2 Optimization

We minimize the disc energy using gradient-descent. Although the disc energy is not convex in its arguments, we found that incorporating Nesterov's[37] momentum factor led to an acceleration of the optimization. One starts with an initial guess of the parameter $\theta_0 = \theta_{-1}$, where θ is a generic variable used to denote the parameters x_c, y_c, and R. The optimization is carried out over positive real numbers. The iterations are given by

$$\theta_{n+1} = \vartheta_n - \gamma_n \nabla E[\theta_n]; \quad E[\theta_0] \geq E[\theta_1] \geq E[\theta_2] \cdots ,$$

where

$$\vartheta_n = \theta_{n-1} + \frac{n-2}{n+1}\left(\theta_{n-1} - \theta_{n-2}\right), n = 1, 2, 3, \cdots$$

The iterates require partial derivatives of the disc energy E with respect to the parameters. Since the integrals are two-dimensional and the contours are closed, one could compute the partial derivatives efficiently using Green's[38] theorem. Applying Green's theorem to E_2 gives

$$E_2 = \oint_{R_2} f^X \mathrm{d}Y = -\oint_{R_2} f^Y \mathrm{d}X, \tag{4}$$

where $f^X(X, Y) = \int_{-\infty}^{X} f(\zeta, Y)\mathrm{d}\zeta$ and $f^Y(X, Y) = \int_{-\infty}^{Y} f(X, \zeta)\mathrm{d}\zeta$. E_2 is a function of (X, Y), which are functions of the parameters of the disc. The partial derivative of E_2 with respect to R is given by

$$\frac{\partial E_2}{\partial R} = \frac{\partial E_2}{\partial X}\frac{\partial X}{\partial R} + \frac{\partial E_2}{\partial Y}\frac{\partial Y}{\partial R}. \tag{5}$$

Substituting (4) in (5) and simplifying gives

$$\begin{aligned}\frac{\partial E_2}{\partial R} &= \oint_{R_2} \frac{\partial f^X}{\partial X}\frac{\partial X}{\partial R}\mathrm{d}Y - \oint_{R_2} \frac{\partial f^Y}{\partial Y}\frac{\partial Y}{\partial R}\mathrm{d}X,\\ \frac{\partial E_2}{\partial R} &= \frac{R}{2}\int_{t=0}^{2\pi} f(X_2, Y_2)\,\mathrm{d}t. \end{aligned} \tag{6}$$

Similarly, applying Green's theorem to E_1 gives

$$E_1 = \oint_{R_1} f^X \mathrm{d}Y = -\oint_{R_1} f^Y \mathrm{d}X. \tag{7}$$

The partial derivative of the energy E_1 with respect to R is given by

$$\frac{\partial E_1}{\partial R} = \frac{\partial E_1}{\partial X}\frac{\partial X}{\partial R} + \frac{\partial E_1}{\partial Y}\frac{\partial Y}{\partial R}. \tag{8}$$

Substituting (7) in (8) and simplifying we get that

$$\begin{aligned}\frac{\partial E_1}{\partial R} &= \oint_{R_1} \frac{\partial f^X}{\partial X}\frac{\partial X}{\partial R}\mathrm{d}Y - \oint_{R_1} \frac{\partial f^Y}{\partial Y}\frac{\partial Y}{\partial R}\mathrm{d}X,\\ \frac{\partial E_1}{\partial R} &= R\int_{t=0}^{2\pi} f(X_1, Y_1)\,\mathrm{d}t. \end{aligned} \tag{9}$$

The partial derivative of the energy with respect to R is

$$\frac{\partial E}{\partial R} = \frac{\partial}{\partial R}\left\{\frac{1}{R^2}(E_1 - 2E_2)\right\} \;=\; \frac{1}{R^2}\left\{\frac{\partial E_1}{\partial R} - 2\frac{\partial E_2}{\partial R}\right\} - \frac{2}{R^3}\left\{E_1 - 2E_2\right\}. \tag{10}$$

Substituting (6) and (9) in (10), and simplifying gives

$$\frac{\partial E}{\partial R} = \frac{1}{R}\left(\int_{t=0}^{2\pi} f(X_1, Y_1)\,\mathrm{d}t - \int_{t=0}^{2\pi} f(X_2, Y_2)\,\mathrm{d}t - 2E\right). \tag{11}$$

Similarly, the partial derivatives of the energy with respect to the coordinates of the center of the disc x_c and y_c can be computed as:

$$\frac{\partial E}{\partial x_c} = \frac{1}{R^2}\left(\int_{t=0}^{2\pi} (\sqrt{2}f(X_1, Y_1)\,\mathrm{d}t - 2f(X_2, Y_2))\cos t\,\mathrm{d}t\right), \tag{12}$$

$$\frac{\partial E}{\partial y_c} = \frac{1}{R^2}\left(\int_{t=0}^{2\pi} (\sqrt{2}f(X_1, Y_1)\,\mathrm{d}t - 2f(X_2, Y_2))\sin t\,\mathrm{d}t\right). \tag{13}$$

S4 Segmentation of Optic Cup

We use Otsu's multilevel thresholding algorithm[39,40] to classify and cluster the pixels corresponding to the optic cup, and then determine the size of the optic cup using circular active disc. Otsu's multilevel thresholding algorithm assumes the image comprising N pixels to be grouped into M classes and calculates optimum thresholds separating classes such that the intra-class variance is minimum. Intra-class variance is the weighted sum of variances of the M classes. Let the grayscale intensities range between 0 and $L-1$ and let f_i be the number of pixels with grey-level i. In a frequentist sense, the probability of the grey-level i is given by $p_i = \frac{f_i}{N}$. The thresholds $1 \leq t_1 < t_2 \cdots < t_{M-1} \leq L$, divide the image into M classes $\{C_1, C_2, ..., C_M\}$. The optimal thresholds can be chosen by maximizing the inter-class variance given by

$$\sigma_B^2 = \sum_{k=1}^{M} \frac{(\mu_k - \mu)^2}{\sum_{i \in C_k} p_i},$$

where μ is the mean intensity of the whole image and

$$\mu_k = \frac{\sum_{i \in C_k} i\, p_i}{\sum_{i \in C_k} p_i}.$$

We found that a four-level thresholding scheme works best for clustering the optic cup pixels. Fig. 4 illustrates the result at the end of each level of thresholding. The corresponding optic cup region is fitted by the circular active disc, the procedure for which has been described in Section S3.

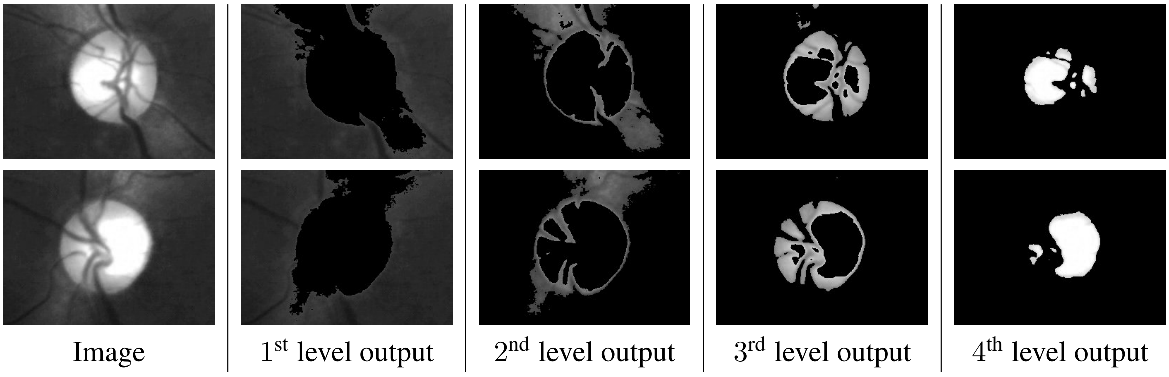

Figure 4: An illustration of the clustering process using Otsu's four-level thresholding scheme. The fourth-level output highlights most of the pixels corresponding to the optic cup.

S5 Screenshots of the iOS App

In the following, we show screenshots of our iOS app in action. We call our app *Nayana* (meaning, “The Eye” in Sanskrit). We have also developed an Android counterpart of the app. The apps are ready for deployment on fundus-on-phone devices.

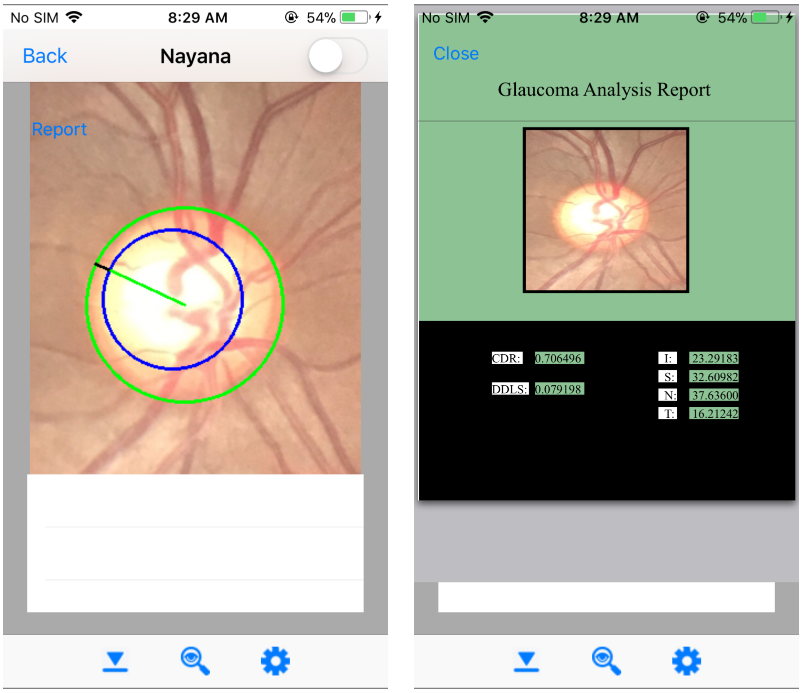

(a) (b)

Figure 5: (a) A screenshot of the iOS app; and (b) An example of a Glaucoma prescreening report generated by the app.

S6 Comparison with the State-of-the-Art:

The algorithm performance has been evaluated on five retinal fundus image databases having images with varied size, resolution, contrast, and illumination. A quantitative comparison of the automatic segmentation of the proposed method and clinician outlining (ground truth) is done using the performance metrics such as sensitivity (Se), specificity (Sp), accuracy (Ac), % error (Pe), Jaccard similarity index (J_I), Dice similarity index (D_I), and time per image (T_I). The performance of the proposed method in comparison with the state-of-the-art OD and OC segmentation techniques is shown in Table 1 and Table 2, respectively. The performance metrics obtained reveal that the algorithm outlining has a higher degree of agreement with the expert outlines over several images randomly selected from five different databases. The average time taken to segment OD and OC in comparison with state-of-the-art techniques is also shown in Table 1 and Table 2, respectively. The results show that the proposed segmentation technique is fast and offers reliable and robust segmentation performance.

Table 1: Optic disc segmentation: Performance comparison of various algorithms.

Algorithm	Database (# images)	Se	Sp	Ac	Pe	J_I	D_I	T_I in seconds
Morales et al.[2]	Drive (40)	—	—-	0.9903	0.0097	0.7163	0.8169	—
	DiaretDB1 (89)	—	—	0.9957	0.0043	0.8173	0.8930	—
	ONHSD (99)	—	—	0.9941	0.0059	0.8045	0.8867	—
	Drions-DB (110)	—	—	0.9934	0.0066	0.8424	0.9084	—
	Messidor (1200)	—	—	0.9949	0.0051	0.8228	0.8950	—
Abdullah et at.[17]	Drive (40)	0.8188	0.9966	0.9672	0.0328	0.7860	0.8722	59.2
	DiaretDB1 (89)	0.8510	0.9984	0.9772	0.0228	0.8510	0.8910	40.0
	ONHSD (99)	0.8857	0.9992	0.9967	0.0033	0.8477	0.9197	65.3
	Drions-DB (110)	0.8508	0.9966	0.9989	0.0011	0.8510	0.9102	43.2
	Messidor (1200)	0.8954	0.9995	0.9989	0.0011	0.8793	0.9339	71.3
Dashtbozorg et al.[41]	ONHSD (89)	—	—	—	—	0.7094	0.8300	—
	Messidor (1200)	—	—	—	—	0.8018	0.8900	—
	INSPIRE-AVR (1200)	—	—	—	—	0.7391	0.8500	—
Kumar et al.[42]	Drishti-GS (101)	—	—	—	—	0.8310	0.9077	—
	Drions-DB (110)	—	—	—	—	0.7212	0.8380	—
	Messidor (1200)	—	—	—	—	0.7325	0.8456	—
Proposed method	Drishti-GS (101)	0.9962	0.9698	0.9732	0.0268	0.8707	0.9309	9.35
	Drions-DB (110)	0.8410	0.9960	0.9850	0.0150	0.8341	0.9095	6.50
	Messidor (1200)	0.8840	0.9894	0.9882	0.0118	0.7858	0.8801	8.44
	Forus (126)	0.9457	0.9957	0.9911	0.0089	0.9319	0.9647	8.12
	Bosch (60)	0.9649	0.9994	0.9968	0.0032	0.9109	0.9534	8.45

The symbol "—" indicates that those values were not reported by the corresponding authors.

Table 2: Optic cup segmentation: Performance comparison of various algorithms.

Algorithm	Database (# images)	Se	Sp	Ac	Pe	J_I	D_I	T_I in seconds
Sivaswamy et al.[43] and Joshi et al.[32]	Drishti-GS (50)	—	—	—	—	0.5873	0.7400	—
Kumar et al.[44]	Drishti-GS (50)	—	—	—	—	0.5749	0.7302	—
	Drions-DB (100)	—	—	—	—	0.5445	0.7050	—
	Messidor (100)	—	—	—	—	0.5527	0.7120	—
Sivaswamy et al.[43] and Joshi et al.[23]	Drishti-GS (50)	—	—	—	—	0.6260	0.7700	—
Proposed method	Drishti-GS (50)	0.8589	0.9698	0.9690	0.0310	0.6143	0.7611	5.46
	Drions-DB (100)	0.6175	0.9979	0.9884	0.0116	0.5898	0.7420	3.54
	Messidor (100)	0.8357	0.9883	0.9823	0.0177	0.5911	0.7430	4.35
	Forus (126)	0.7466	0.9952	0.9867	0.0133	0.6801	0.8096	4.12
	Bosch (60)	0.7584	0.9959	0.9917	0.0083	0.5804	0.7345	4.28

The symbol "—" indicates that those values were not reported by the corresponding authors.

References

[1] Foracchia, M., Grisan, E. & Ruggeri, A. Detection of optic disc in retinal images by means of a geometrical model of vessel structure. *IEEE Transactions on Medical Imaging* **23**, 1189–1195 (2004).

[2] Morales, S., Naranjo, V., Angulo, J. & Alcaiz, M. Automatic detection of optic disc based on pca and mathematical morphology. *IEEE Transactions on Medical Imaging* **32**, 786–796 (2013).

[3] Carmona, E. J., Rincn, M., Garca-Feijoo, J. & de-la Casa, J. M. M. Identification of the optic nerve head with genetic algorithms. *Artificial Intelligence in Medicine* **43(3)**, 243–259 (2008).

[4] Mahfouz, A. E. & Fahmy, A. S. Fast localization of the optic disc using projection of image features. *IEEE Transactions on Image Processing* **19**, 3285–3289 (2010).

[5] Aquino, A., Gegundez-Arias, M. E. & Marin, D. Detecting the optic disc boundary in digital fundus images using morphological, edge detection, and feature extraction techniques. *IEEE Transactions on Medical Imaging* **29**, 1860–1869 (2010).

[6] Yu, H. *et al.* Fast localization and segmentation of optic disk in retinal images using directional matched filtering and level sets. *IEEE Transactions on Information Technology in Biomedicine* **16**, 644–657 (2012).

[7] Giachetti, A., Ballerini, L. & Trucco, E. Accurate and reliable segmentation of the optic disc in digital fundus images. *J. Med. Imag.* **1(2)**, 1–11 (2014).

[8] Youssif, A. A. H. A. R., Ghalwash, A. Z. & Ghoneim, A. A. S. A. R. Optic disc detection from normalized digital fundus images by means of a vessels' direction matched filter. *IEEE Transactions on Medical Imaging* **27**, 11–18 (2008).

[9] Joshi, G. D., Gautam, R., Krishnadas, S. R. & Sivaswamy, J. Robust optic disk segmentation from colour retinal images. In *Proc. Intl. Conf. on Vision Graphics and Image Processing (ICVGIP)*, 330–336 (2010).

[10] Muramatsu, C. *et al.* Automated segmentation of optic disc region on retinal fundus photographs: Comparison of contour modelling and pixel classification methods. *Computer Methods and Programs in Biomedicine, Elsevier* **101**, 23–32 (2011).

[11] Lowell, J. *et al.* Optic nerve head segmentation. *IEEE Transactions on Medical Imaging* **23**, 256–264 (2004).

[12] Pallawala, P., Hsu, W., Lee, M. & Eong, K. Automated optic disc localization and contour detection using ellipse fitting and wavelet transform. In *Proc. Europian Conference on Computer Vision (ECCV)*, 139–151 (2004).

[13] Chrastek, R., Wolf, M., Donath, K., Michelson, G. & Niemann, H. Optic disc segmentation in retinal images. *Bildverarbeitung für die Medizin* 263–266 (2002).

[14] Welfer, D. *et al.* Segmentation of the optic disk in color eye fundus images using an adaptive morphological approach. *Computers in Biology and Medicine, Elsevier* **40**, 124–137 (2010).

[15] Osareh, A. *Automated Identification of Diabetic Retinal Exudates and the Optic Disc*. Ph.D. thesis, Department of Computer Science, University of Bristol (2004).

[16] Walter, T., Klein, J. C., Massin, P. & Erginay, A. A contribution of image processing to the diagnosis of diabetic retinopathy-detection of exudates in color fundus images of the human retina. *IEEE Transactions on Medical Imaging* **21**, 1236–1243 (2002).

[17] Abdullah, M., Fraz, M. M. & Barman, S. A. Localization and segmentation of optic disc in retinal images using circular hough transform and grow-cut algorithm. *PeerJ* **4:e2003**, 1/22–22/22 (2016).

[18] S-Gonzalez, A., Kaba, D., Li, Y. & Liu, X. Segmentation of the blood vessels and optic disk in retinal images. *IEEE Journal of Biomedical and Health Informatics* **18**, 1874–1886 (2014).

[19] Sevastopolsky, A. Optic disc and cup segmentation methods for glaucoma detection with modification of U-Net convolutional neural network. *Pattern Recognition and Image Analysis* **27**, 618–624 (2017).

[20] Ronneberger, O., Fischer, P. & Brox, T. U-net: Convolutional networks for biomedical image segmentation. In *Proc. International Conference on Medical Image Computing and Computer-Assisted Intervention*, 234–241 (Springer, 2015).

[21] Maninis, K.-K., Pont-Tuset, J., Arbeláez, P. & Van Gool, L. Deep retinal image understanding. In *Proc. International Conference on Medical Image Computing and Computer-Assisted Intervention*, 140–148 (Springer, 2016).

[22] Simonyan, K. & Zisserman, A. Very deep convolutional networks for large-scale image recognition. *CoRR* **abs/1409.1556** (2014).

[23] Joshi, G. D., Sivaswamy, J. & Krishnadas, S. R. Depth discontinuity-based cup segmentation from multiview color retinal images. *IEEE Transactions on Biomedical Engineering* **59**, 1523–1531 (2012).

[24] Joshi, G. D., Sivaswamy, J., Karan, K., Prashanth, R. & Krishnadas, S. R. Vessel bend-based cup segmentation in retinal images. In *Proc. Intl. Conf. on Pattern Recognition (ICPR)*, 2536–2539 (2010).

[25] Hatanaka, Y. *et al.* Improved automated optic cup segmentation based on detection of blood vessel bends in retinal fundus images. In *Proc. 36th Annual International Conference of the IEEE Engineering in Medicine and Biology Society (EMBC)*, 126–129 (2014).

[26] Chakravarty, A. & Sivaswamy, J. Coupled sparse dictionary for depth-based cup segmentation from single color fundus image. In *Proc. Intl. Conf. on Medical Image Computing and Computer-Assisted Intervention (MICCAI)*, 747–754 (2014).

[27] Xu, Y. *et al.* Optic cup segmentation for glaucoma detection using low-rank superpixel representation. In *Proc. Intl. Conf. on Medical Image Computing and Computer-Assisted Intervention (MICCAI)*, 788–795 (2014).

[28] Luangruangrong, W., Chinnasarn, K. & Rodtook, A. Polar space contour detection for automated optic cup segmentation. In *2018 10th International Conference on Knowledge and Smart Technology (KST)*, 164–169 (2018).

[29] Cheng, J. *et al.* Superpixel classification based optic disc and optic cup segmentation for glaucoma screening. *IEEE Transactions on Medical Imaging* **32**, 1019–1032 (2013).

[30] Xu, J., Chutatape, O., Sung, E., Zheng, C. & Kuan, P. C. T. Optic disk feature extraction via modified deformable model technique for glaucoma analysis. *Pattern Recognition* **40**, 2063 – 2076 (2007).

[31] Joshi, G. D., Sivaswamy, J., Karan, K. & Krishnadas, S. R. Optic disk and cup boundary detection using regional information. In *Proc. IEEE Intl. Symposium on Biomedical Imaging: From Nano to Macro (ISBI)*, 948–951 (2010).

[32] Joshi, G. D., Sivaswamy, J. & Krishnadas, S. R. Optic disk and cup segmentation from monocular color retinal images for glaucoma assessment. *IEEE Transactions on Medical Imaging* **30**, 1192–1205 (2011).

[33] Yin, F. *et al.* Automated segmentation of optic disc and optic cup in fundus images for glaucoma diagnosis. In *2012 25th IEEE International Symposium on Computer-Based Medical Systems (CBMS)*, 1–6 (2012).

[34] Zahoor, M. N. & Fraz, M. M. A correction to the article "fast optic disc segmentation in retina using polar transform". *IEEE Access* **6**, 4845–4849 (2018).

[35] Cheng, J. *et al.* Structure-preserving guided retinal image filtering and its application for optic disc analysis. *IEEE Transactions on Medical Imaging* (accepted for publication) (2018).

[36] Winder, R. J., Morrow, P. J., McRitchie, I. N., Bailie, J. R. & Hart, P. M. Algorithms for digital image processing in diabetic retinopathy. *Comput. Med. Imaging. Graph* **33(8)**, 608–622 (2009).

[37] Nesterov, Y. *Introductory Lectures on Convex Optimization: A Basic Course* (Springer, USA, 2004). URL `http://www.ece.osu.edu/~randy/SAtext`.

[38] Simmons, G. F. *Calculus With Analytic Geometry* (The McGraw-Hill Companies, Inc., USA, 1996). URL `http://www.ece.osu.edu/~randy/SAtext`.

[39] Otsu, N. A threshold selection method from gray-level histograms. *IEEE Transactions on Systems, Man, and Cybernetics* **9(1)**, 62–66 (1979).

[40] Liao, P., Chen, T. & Chung, P. A fast algorithm for multilevel thresholding. *Journal of Information Science and Engineering* **17**, 713–727 (2001).

[41] Dashtbozorg, B., Mendonca, A. M. & Campilho, A. Optic disc segmentation using the sliding band filter. *Computers in biology and medicine, Elsevier* **56**, 1–12 (2014).

[42] Kumar, J. R. H., Pediredla, A. K. & Seelamantula, C. S. Active discs for automated optic disc segmentation. In *IEEE Global Conference on Signal and Information Processing (GlobalSIP)*, 225–229 (2015).

[43] Sivaswamy, J. *et al.* A comprehensive retinal image dataset for the assessment of glaucoma from the optic nerve head analysis. *JSM Biomedical Imaging* **9**, 1–7 (2015).

[44] Kumar, J. R. H., Harsha, S., Kamath, Y., Jampala, R. & Seelamantula, C. S. Automatic optic cup segmentation using kåsa's circle fitting technique. In *IEEE Region 10 Conference (TENCON 2017)*, 25–30 (2017).
